# Contraceptive uptake after training community health workers in couples counseling: A cluster randomized trial

**DOI:** 10.1371/journal.pone.0175879

**Published:** 2017-04-27

**Authors:** Clara Lemani, Jennifer H. Tang, Dawn Kopp, Billy Phiri, Chrissy Kumvula, Loyce Chikosi, Mwawi Mwale, Nora E. Rosenberg

**Affiliations:** 1 UNC Project-Malawi, Lilongwe, Malawi; 2 University of North Carolina at Chapel Hill, School of Medicine, Department of Obstetrics & Gynecology, Chapel Hill, North Carolina, United States of America; 3 Area 25 Health Centre, Lilongwe, Malawi; 4 Lilongwe District Health Office, Lilongwe, Malawi; 5 University of North Carolina at Chapel Hill, School of Public Health, Department of Epidemiology, Chapel Hill, North Carolina, United States of America; University of Pittsburgh, UNITED STATES

## Abstract

**Background:**

Young women in Malawi face many challenges in accessing family planning (FP), including distance to the health facility and partner disapproval. Our primary objective was to assess if training HSAs in couples counseling would increase modern FP uptake among young women.

**Methods:**

In this cluster randomized controlled trial, 30 HSAs from Lilongwe, Malawi received training in FP. The HSAs were then randomized 1:1 to receive or not receive additional training in couples counseling. All HSAs were asked to provide FP counseling to women in their communities and record their contraceptive uptake over 6 months. Sexually-active women <30 years of age who had never used a modern FP method were included in this analysis. Generalized estimating equations with an exchangeable correlation matrix to account for clustering by HSA were used to estimate risk differences (RDs) and 95% confidence intervals (CIs).

**Results:**

430 (53%) young women were counseled by the 15 HSAs who received couples counseling training, and 378 (47%) were counseled by the 15 HSAs who did not. 115 (26%) from the couples counseling group had male partners present during their first visit, compared to only 6 (2%) from the other group (RD: 0.21, 95% CI: 0.09 to 0.33, p<0.01). Nearly all (99.5%) initiated a modern FP method, with no difference between groups (p = 0.09). Women in the couples counseling group were 8% more likely to receive male condoms (RD: 0.08, 95% CI: -7% to 23%, p = 0.28) and 8% more likely to receive dual methods (RD: 0.08, 95% CI: -0.065, 0.232, p = 0.274).

**Conclusion:**

Training HSAs in FP led to high modern FP uptake among young women who had never used FP. Couples counseling training increased male involvement with a trend towards higher male condom uptake.

## Introduction

High unmet need for family planning (FP) contributes to low contraceptive use, especially among young women. The total unmet need for FP in Malawi among reproductive age women is estimated at 18.5%: 10.2% for spacing and 8.2% for limiting [1]. For young women (aged less than 30 years), unmet need for FP is even higher at 25% [2]. Addressing this unmet need for FP is crucial in the formulation of policies and programs aimed at increasing contraceptive uptake and continuation rates.

Contraceptive uptake in Malawi is considerably low. Poor access to the nearest clinic for FP and lack of availability of the desired method contribute to low FP uptake [3]. In order to increase access to FP and other reproductive health services, studies have recommended using community-based interventions targeting young married couples [4, 5]. Many countries have therefore started training community health workers in short-term FP method provision to reach rural and underserved women [6, 7]. In Malawi, community-based distribution of family planning has been available since 2010, when Malawi implemented a pilot program on provision of the depot medroxyprogesterone acetate (DMPA) injectable by a group of community health workers called Health Surveillance Assistants (HSAs). The pilot program found that over 75% of HSA DMPA clients interviewed felt that people in the community approved of the program, and over 90% said that they were very satisfied with the counseling and information they received from the HSA [7]. Since then, Malawi has been scaling-up the use of HSAs around the country to provide FP services in the community. However, HSAs are not allowed to provide contraceptive methods until they undergo a 1-week training in FP [8].

Studies have explored factors contributing to high unmet need for FP. These factors include lack of male involvement in FP, myths and misconceptions about FP, poor access to health facilities, lack of availability of FP methods at health facilities, and poor awareness and knowledge of FP [9, 10]. Studies in Malawi and India have demonstrated the benefits of involving men in community-based interventions on FP and other reproductive health programs [11, 12]. In some settings, men are considered as the head of the household, and they often need to approve any decision being made at household level, including FP use [13]. Including men in discussions about FP may therefore help to increase FP uptake and continuation among women because if men have the correct information about FP and its benefits, they may be more likely to support their partner’s use of it. In addition, including men could also encourage spousal discussion about FP, leading to a mutual decision on which method to use [14].

Given the success and acceptability of the HSA program in Malawi and the need to include male partners in FP decision-making, we designed a cluster randomized trial in which HSAs at Area 25 Health Centre in Lilongwe, Malawi, were randomized to receive or not receive training in couples counseling for FP. Our primary objective was to assess if young women counseled by HSAs trained in couples counseling were more likely to initiate modern FP methods than young women counseled by HSAs who were not trained in couples counseling. Our hypothesis was that women counseled by HSAs who received couples counseling training would be more likely to initiate a modern FP method than those counseled by HSAs who did not. Secondary objectives included evaluating whether young women counseled by HSAs trained in couples counseling would be more likely to use long-term FP methods (implant, intrauterine device (IUD), female or male sterilization), continue use of hormonal or intrauterine methods 6 months after contraceptive initiation, and use dual methods of contraception (both a modern FP method and a barrier method).

## Methods

### Study design and population

This study was approved by the UNC IRB (#13–3338) and Malawi National Health Research Committee (#1210). The study engaged 30 HSAs from Area 25 Health Centre, a peri-urban primary-level health center located about 15 km north of Lilongwe, Malawi’s capital. HSAs in Malawi can be either male or female but must have completed 12 years of education and a Malawi School Certificate of Education and be from their catchment area [15]. They are employed by the Government and attached to a hospital or health center but spend most of their time in the community. Currently, they receive 12 weeks of basic training to provide community-level primary health care services, including immunizations, health education, and community assessment and mobilization [15, 16]. They may then complete an additional week of training in FP, during which they learn about all available contraceptives in Malawi and are trained to directly provide condoms, oral contraceptives, and DMPA and refer for other methods.

All the HSAs in our program underwent the 1-week Malawi Ministry of Health (MoH)-approved HSA FP training in November 2014 and received a bicycle so that they could more easily travel to their catchment areas. The HSAs were then stratified by sex and catchment area (urban versus rural) to ensure that these two potential confounders were evenly-distributed between the two randomized groups. Each strata was then randomized to receive or not to receive an additional 2-day couples counseling training in February 2015.

The couples counseling training was adapted from Malawi’s national training on couple HIV testing and counseling and Save the Children’s Male Motivator curriculum [17] and gave participants an opportunity to discuss Malawian gender norms, as well as FP benefits for men, women and infants. It contained didactic instruction on communication and couple counseling techniques and practical sessions on applying these techniques to couples with FP challenges. The training also encouraged contraceptive choice, but emphasized condoms plus another method to maximize both pregnancy prevention, as well as protection from HIV and STIs. No refresher trainings were held after the initial training period.

After the trainings, all of the HSAs were asked to visit women from their catchment areas at their homes and counsel them on FP. For those HSAs who received the additional training in couples counseling, they were also asked to offer their clients counseling with the male partner present if the female client was interested. The HSA would then initiate the method of the woman’s choice if the woman asked for a short-term method or refer to the nearest facility for long-term methods. They then received an ~$1 incentive for each woman that they counseled that was eligible for our study, for up to a maximum of 30 women over 4 months of recruitment (March-June 2015). The standard demographic and FP information from each visit was recorded in the Malawi MoH-approved HSA FP Registers. Information from the eligible women recorded in the registers was then entered into a study database.

To be eligible for inclusion in the study, the counseled women must have met the following eligibility criteria: (a) less than 30 years old, (b) had vaginal intercourse at least once in past 3 months, (c) have a male partner, (d) had never used any modern FP method (oral contraceptives, injection, implant, IUD, or sterilization). All eligible clients were followed up by the HSA’s 3 months and 6 months after their initial HSA FP visit, and their follow-up information was also recorded in the FP Registers. This study used data that is routinely collected by the HSAs; no individual consent was sought from the participants. The HSAs are, however, required to get verbal consent from the women for FP counseling. In addition, our database did not include any identifiable information, such as the name of the participants or their address. We received Malawi and UNC IRB approval to abstract the routine family planning data from the HSA Registers and enter it into our de-identified database.

### Statistical analysis

A study database in Microsoft Access 2003 [18] was created for data entry and management. Data were then exported to STATA version 10 (StataCorp LLP, College Station, TX, USA) for statistical analysis. Descriptive statistics were calculated to report the proportion of respondents in different categorical variables. The main outcomes were: male partner present during counseling, whether a contraceptive method was given during the visit, receipt of condoms, dual method use, and referral to health center for long-acting methods. The exposure was whether or not the HSA had received couples counseling training; women who received FP counseling from HSAs in the couples counseling training were categorized in the “couples counseling group”, whereas women who received FP counseling from HSAs who were not randomized to receive couples counseling training were categorized in the “routine counseling group”. To account for clustering by HSA, generalized estimating equations with an exchangeable correlation matrix were used. We calculated risk differences between the two arms. These risk differences were used to determine whether there was a difference in modern FP uptake, short-term FP uptake, long-term FP uptake, receipt of condoms, and presence of the male partner during counseling between the two randomized groups.

## Results

Of the 30 HSAs, 6 were male, and 3 were randomized to each group (Table 1). Similarly, the 10 HSAs with urban catchment areas were evenly divided between the two groups. The mean number of participants enrolled per HSA in the couples counseling group (28.6; min 22, max 31), was not significantly different from the mean number enrolled per HSA in the routine counseling group (25.2; min 0, max 30) (p = 0.35). One HSA became uninterested in participating in our program after the FP training and being randomized, and therefore did not recruit any participants.

**Table 1:** Health Surveillance Assistant and family planning client characteristics.

**Health Surveillance Assistant (N = 30)**	**Couples Counseling Group (n = 15)**	**Routine Counseling Group (n = 15)**
**Gender/Catchment area of Health Surveillance Assistant**		
Male/Rural	3 (20%)	3 (20%)
Female/Rural	7 (47%)	7 (47%)
Female/Urban	5 (33%)	5 (33%)
**Age of Health Surveillance Assistant**		
20–29 years	1 (6%)	1 (6%)
30–39 years	6 (40%)	7 (47%)
40–49 years	8 (54%)	7 (47%)
**Number of participants recruited by each Health Surveillance Assistant (mean, standard deviation)**	28.6 (2.84)	25.2 (8.35)
**Clients (N = 808)**	**Couples Counseling Group (n = 430)**	**Routine Counseling Group (n = 378)**
**Age of client**		
14–19 years	96 (22%)	96 (26%)
20–25 years	198 (46%)	179 (47%)
26–30 years	136 (32%)	103 (27%)
**Residence**		
Rural	291 (68%)	251 (66%)
Urban	139 (32%)	127 (34%)
**Marital status**		
Not Married	44 (10%)	67(18%)
Married	386 (90%)	311 (82%)
**Number of living children**		
No child	13 (3%)	34 (9%)
1 child	214 (50%)	225 (60%)
2 children	130 (30%)	70 (19%)
3 children	73 (17%)	49 (13%)
**Number of clients seen, by sex of Health Surveillance Assistant**		
Female	341 (79%)	288 (76%)
Male	89 (21%)	90 (24%)

808 women were enrolled in the study, 430 (53%) in the couples counseling group and 378 (47%) in the routine counseling group. In both groups, most women were between 20 and 25 years (46% in the couples counseling group, 47% in the routine counseling group). The median age (22 years) and interquartile range (5 years) were the same in both groups. The majority of women in both groups were from the rural areas (68% and 66% in the couples counseling and routine counseling groups, respectively). Thirty-four women (9%) in the routine counseling group had no living children as compared to 13 (3%) in the couples counseling group.

Most women reported being married (90% in the couples counseling group and 82% in the routine counseling group). 27% of women in the couples counseling group and 6% in the routine counseling group had their partners present during FP counseling at the baseline visit, which was a significant difference (RD: 0.21, 95% CI: 0.09 to 0.33, p<0.01) (Table 2).

**Table 2:** Family planning outcomes at baseline, 3-month and 6-month follow-up visits.

	Baseline visit		3-month follow-up visit		6-month follow-up visit	
	Couples Counseling Group (n = 430)	Routine Counseling Group (n = 378)	Couples Counseling Group (n = 421)	Routine Counseling Group (n = 365)	Couples Counseling Group (n = 420)	Routine Counseling Group (n = 361)
Male partner present	115 (27%)	6 (2%)	13 (3%)	2 (0.6%)	0 (0%)	0 (0%)
FP method given						
OC	36 (8%)	27 (7%)	21 (5%)	21 (6%)	21 (5%)	17 (5%)
DMPA	386 (90%)	347 (92%)	390 (92%)	338 (93%)	387 (92%)	338 (93%)
Did not receive OC or DMPA	8 (2%)	4 (1%)	10 (2%)	6 (2%)	12 (3%)	6 (2%)
Received Condoms	180 (42%)	115 (30%)	2 (0.5%)	0 (0%)	28 (7%)	10 (3%)
Referred for FP	31 (7%)	14 (4%)	3 (0.7%)	0 (0%)	1 (0.24%)	3 (0.8%)
Implant	29 (93%)	11 (79%)	3 (100%)	0 (0%)	1 (100%)	0
IUD	1 (3%)	1 (7%)	0 (0%)	0 (0%)	0 (0%)	0
BTL	1 (3%)	2 (14%)	0 (0%)	0 (0%)	0 (0%)	1
Vasectomy	0	0	0	0	0	2
Went for FP referral						
Yes	10 (32%)	6 (43%)	1 (33%)	0 (0%)	N/A	N/A
Implant	10	6	1	0		
IUD	0	0	0	0		
BTL	0	0	0	0		
No	20 (65%)	8 (57%)	2 (67%)	N/A	N/A	N/A
Implant	18	5	2			
IUD	1	1	0			
BTL	1	2	0			

FP, Family Planning; OC, oral contraceptives; DMPA, Depot medroxyprogesterone acetate; IUD, intrauterine device; BTL, bilateral tubal ligation

Most women in both groups initiated a FP method at the first visit (99% in the routine counseling group and 98% in the couples counseling group, p = 0.22). (Table 2). The most common method given at this visit was DMPA, with 90% in the couples counseling group and 92% in routine counseling group receiving this method. FP continuation was high: more than 90% of those followed during the 3-month and 6-month follow-up visits received a FP method during the follow-up visit. However, there was no significant difference in continuation at 6 months between women in the couples counseling (97.4%) and women in the routine counseling group (94.2%) (RD: 0.033, 95% CI: -0.001 to 0.068, p = 0.064) (Table 3).

**Table 3:** Summary statistics for family planning outcomes.

Family Planning Outcome	Couples Counseling Group	Routine Counseling Group	Risk Difference	P- value	Confidence Intervals
Started Modern FP	426 (99.1%)	378 (100%)	-0.009	0.087	-0.020, 0.001
Started LARC	11 (2.6%)	6 (1.6%)	0.010	0.458	-0.016, 0.037
Started SARC	415 (97.4%)	372 (98.4%)	-0.010	0.443	-0.038, 0.016
FP Method switch	20 (4.8%)	7 (1.9%)	0.015	0.372	-0.017, 0.048
Continuation of FP method at 6 months	419 (97.4%)	356 (94.2%)	0.033	0.064	-0.001, 0.068
Received dual method	176 (61.3%)	111 (38.6%)	0.083	0.274	-0.065, 0.232

FP, family planning; LARC, long acting reversible contraception; SARC, short acting reversible contraception

Women who preferred a long-term method were referred to the nearest health facility where they could access the method. At the baseline visit, 31 (7%) and 14 (4%) of women from the couples counseling and routine counseling groups, respectively, were referred for long-term methods, the majority (89%) for implant. Of those referred, 10 (32%) in the couples counseling and 6 (43%) in routine counseling group went and received a long-term method at a health facility. 3 women were referred during the 3-month follow-up visit; only 1 went and received the method. All women who received LARC were still using at the 6-month follow-up visit.

After counseling, women were also offered male condoms. One hundred eighty women (42%) in the couples counseling group and 115 (30%) in routine counseling group received male condoms at baseline (RD: 0.08, 95% CI: -7% to 23%, p = 0.20). During the 6-month follow-up visit, 28 women (7%) in the couples counseling group and 10 (3%) in the routine counseling group received male condoms. This indicates an increase from the 3-month follow-up visit, during which only 2 women (0.5%) in the couples counseling group received male condoms. Couples counseling emphasized dual method use, but we found no difference in the provision of dual methods among women between the two groups of HSAs (RD: 0.083, 95% CI: -0.065, 0.232, p = 0.274). We also looked at male condom provision among women who had their male partners present during counseling, regardless of whether they were counseled by HSAs who received couples counseling or not. There was a significant association as 45% of those who had their male partner present received male condoms, compared to 35% of those who did not have their male partner present (p-value = 0.044).

## Discussion

This study assessed if training HSAs in couples counseling has an added impact on modern FP uptake and continuation six months after initiation among young women. The findings showed no significant difference in modern FP uptake among young women counseled by HSAs who received routine counseling training and those who received couples counseling training as nearly all women in both groups initiated a FP method. Women counseled by HSAs who received couples counseling training were more likely to have their partners present during counseling and to receive condoms at their first visit. There was no difference in the proportion of women who initiated short-acting or long-acting methods between the two groups, or in those who continued using hormonal or intrauterine methods at the 6-month follow-up visit.

FP uptake was high in both groups as more than 98% of women initiated a modern FP method. These results however should be interpreted with caution as this is only among women who consented for FP counseling. The HSAs did not capture any information on those who refused to be counseled on FP. The results, however, add to the evidence that community-based FP interventions can lead to high contraceptive uptake among women. A similar study in Bangladesh which offered door-to-door counseling and distribution of contraceptives showed large statistically significant and enduring increases in uptake of contraceptives in the intervention area [19]. Another randomized controlled trial in Jordan using community health workers to provide home-based FP services to women and couples also found a substantial increase in uptake of modern contraceptives [6]. These findings show the importance of community-based FP service delivery for women to better learn about the methods and dispel the myths perceived to be associated with contraceptive use.

Our findings showed no significant difference in FP uptake between those counseled by HSAs who received couples counseling versus routine counseling. This is contrary to the notion that couples counseling leads to high modern FP uptake as it leads to increased spousal communication, which could then lead to increased knowledge and comfort in communicating FP issues [20, 21]. Our results are, however, similar to results of studies done in Jordan and Zambia, which found that the effect of couples versus individual counseling was statistically not significant [6, 22]. The lack of difference in modern FP uptake in our study may be secondary to the fact that only those who agreed to FP counseling were included in our study, which may imply that they already had an interest in FP use, which then led to high FP uptake. This finding also could be due to low participation of men in FP counseling sessions, which led to very low vasectomy uptake among men in the study, though this low uptake is consistent with national data [1]. Absence of husband at home during the counseling and refusal have been identified as the reasons men are rarely counseled about FP [6]. This study, however, did not document why men were not present during counseling. A study in Malawi in which men were actively involved showed a significant increase in FP uptake. This randomized control study recruited men whose partners were not using any contraception and randomized them to receive FP counseling from a male motivator or not. Their FP uptake was then recorded after the intervention, and 78% in the intervention arm were using FP as compared to 58% in the control arm (p<0.01) (23). Another study in India demonstrated the effectiveness of engaging husbands in interventions aimed at improving contraceptive use among young couples [6].

A majority of women who initiated a family planning method chose a short-acting reversible contraceptive (SARC), especially DMPA. Among those who initiated a FP method, only 2.2% chose implant and 0.1% chose IUD. This may be because within this study it was easier to get SARC in the community than to go to the clinic to get long acting reversible method (LARC). The findings however are consistent with data from the 2010 Malawi Demographic and Health Survey which shows that most women of reproductive age who were using a contraceptive method at the time of the survey were using DMPA. In the same survey, more women reported using implant (1.1%) than IUD (0.2%). A study in Zambia also showed lower uptake of long acting reversible contraception (LARC) as compared to SARC and for the LARC, implant was preferred to IUD [22]. Among other reasons, LARC uptake is underused due to women’s and providers misperceptions and misinformation; and lack of skilled staff at health facilities[23]. Community mobilization, staff training and ensuring continuous supply of the LARC commodities may help increase LARC uptake.

Continuation of hormonal and intrauterine methods at 3 and 6 months was high among women who initiated them in this study. More than 90% of women in both groups continued with their hormonal or intrauterine method. The number of discontinuers includes those who were not reached at both follow-up visits as their FP use could not be recorded, so our actual discontinuation estimate may be lower than we calculated if some women had received their FP method elsewhere. Women were allowed to choose their method at each visit, and some chose a method different from that received at baseline, and some who chose not to receive a method at baseline received one during the follow-up visits. Most of those who switched methods switched from using SARC to LARC, specifically implants. FP continuation in this study was higher than that found in a similar study in Zambia, in which couples were randomized in one of the three family planning education videos. They found only a 50% continuation rate among women who were followed at 3 months [22].

Our study also investigated condom distribution. We evaluated whether women counseled by HSAs in the couples counseling group were more likely to receive and use condoms for prevention of sexually transmitted diseases, including HIV. More women in the couples counseling group received condoms as compared to those in the routine counseling group at baseline, although less than half of the participants in both groups received condoms at any visit. In addition, there was significant difference in condom receipt among those who had their male partners present during counseling and those who did not, which suggests that couples more likely to receive condoms when the male partner is present. However, data on actual condom usage as reported by the women had too many inconsistences and missing data so could not be analyzed. In addition, condom distribution during the 3-month follow-up visit was affected by condom stock-outs at Area 25 Health Center during that study period, and as a result, only 2 (0.5%) women in the couples counseling group received condoms during this visit.

This study used a predesigned standard register for data collection. This was done to reduce workload for the HSAs and also minimize errors. However, use of the standard register was also a limitation as we could not collect additional information; for example, reasons why men were not present during counseling. Other strengths of our study include our ability to target the entire catchment area for a peri-urban health center and our low loss-to-follow-up rate. Weaknesses also included our inability to ensure a constant supply of male condoms at the health center (which affected our 3-month follow-up condom uptake), our inability to verify if the women were truly new users of FP, and the lack of documentation of the number of women who refused FP counseling.

## Conclusion

Our results suggest that training and incentivizing HSAs to provide FP in the communities led to nearly universal uptake of modern FP among young women. Young women in Malawi have high unmet need for FP; adopting community-based interventions like ours may assist in reducing it. Couples counseling did not have an added impact on FP uptake in this population of women, but it led to increased male involvement and a trend towards higher condom uptake. Condom usage may help reduce transmission of STIs and HIV; further research could therefore look at the actual use of condoms among those FP clients who accept condoms and reasons why men do not attend home-based FP visits.

## Supporting information

S1 File HSA study data. (DTA)

## References

[1] National Statistics Office (NSO) and ICF Macro, Malawi Demographic and Health Survey. Zomba, Malawi and and Calverton, Maryland, USA: NSO and ICF Macro, 2011.

[2] MacQuarrie KLD. Unmet Need for Family Planning among Young Women: Levels and Trends. Rockville, Maryland, USA: ICF International, 2014.

[3] Megquier S. Reproductive Transitions: Unmet Need for Family Planning. Population Reference Bureau, 2014.

[4] Sarkar A, Chandra-Mouli V, Jain K, Behera J, Mishra S K, Mehra S. Community based reproductive health interventions for young married couples in resource-constrained settings: a systematic review. BMC Public Health. 2015;15(1037). 10.1186/s12889-015-2352-7 PMC4599316 26452750

[5] Daniel EE, Masilamani R, Rahman M. The Effect of Community-Based Reproductive Health Communication Interventions on Contraceptive Use Among Young Married Couples in Bihar,India. International Family Planning Prespectives. 2008;34(4):189–97. 10.1363/ifpp.34.189.08 19201679

[6] Abt Associates. Counseling Women and Couples in Family Planning: Evidence from Jordan. Bethesda, MD 20814 USA: 2015.

[7] FHI360. Evaluation of Community-Based Distribution of DMPA by Health Surveillance Assistants in Malawi. NC 27709 USA: FHI, 2010.

[8] Richardson F, Chirwa M, Fahnestock M, Bishop M, Emmart P, McHenry B. Community-based Distribution of Injectable Contraceptives in Malawi. Washington, DC: Futures Group International, 2009.

[9] Gueye A, Speizer IS, Corroon M, Okigbo H.C. Belief in Family Planning Myths at the Individual s and Modern Contraceptive Use in Urban Africa. Int Perspect Sex Reprod Health. 2015;41(4):191–9. doi: 10.1363/4119115 26871727 10.1363/4119115 PMC4858446

[10] Ankomah A, Anyati J, Oladosu M. Myths, misinformation, and communication about family planning and contraceptive use in Nigeria. Journal of Contraception. 2011(2):95–105.

[11] Becker S, Taulo FO, Hindin M J, Chipeta E K, Loll D, Tsui A. Pilot study of home-based delivery of HIV testing and counseling and contraceptive services to couples in Malawi. BMC Public Health. 2014;14(1309). 10.1186/1471-2458-14-1309 PMC4320475 25526799

[12] Raj A, Ghule M, Ritter J, Battala M, Gajanan V, Nair S, et al Cluster Randomized Controlled Trial Evaluation of a Gender Equity and Family Planning Intervention for Married Men and Couples in Rural India. PLoS ONE. 2015;11(5). 10.1371/journal.pone.0153190 PMC4864357 27167981

[13] Darteh EKM, Doku D, Esia-Donkoh K. Reproductive health decision making among Ghanaian women. Reproductive health. 2014;11(23). 10.1186/1742-4755-11-23 PMC3985591 24628727

[14] FHI 360. Increasing Male Involvement in Family Planning in Jharkhand, India. NC 27709 USA: FHI 360, 2013.

[15] Advancing Partners Communities. Country Profile: Malawi Community Health Programs. Arlington, VA: Advancing Partners & Communities, 2014.

[16] Nyirenda L, Namakhoma I, Chikaphupha K, Kok MC, Theobald S. Report on the context analysis of close-to-community providers in Malawi. Lilongwe: REACH Trust, 2014.

[17] Shattuck D, Kerner B, Gilles K, Hartmann M, Ng’ombe T, Guest G. Encouraging contraceptive uptake by motivating men to communicate about family planning: The Malawi Male Motivator project. American Journal of Public Health. 2011;101(6). 10.2105/AJPH.2010.300091 PMC3093271 21493931

[18] MicrosoftCorporation. Microsoft Access. Redmond, WA, USA: Microsoft, 2010.

[19] Phillips JF, Hossain MB. The Impact of Household Delivery of Family Planning Services on Women’s Status in Bangladesh. International Family Planning Prespectives. 2003;29(3):138–45. 10.1363/ifpp.29.138.03 14519591

[20] El-Khoury M, Thornton R, Chatterji M, Kamhawi S, Choi S. Couples vs. Individual Family Planning Counseling: A Randomized Experiment

[21] Tilahun T, Coeneg G, Temmerman M, Degomme O. Couple based family planning education: changes in male involvement and contraceptive use among married couples in Jimma Zone, Ethiopia. BMC Public Health. 2015;15(682). 10.1186/s12889-015-2057-y PMC4509724 26194476

[22] Haddad L, Wall K, Vwalika B, Khu NH, Brill I, Kilembe W, et al Contraceptive discontinuation and switching among couples receiving integrated HIV and family planning services in Lusaka, Zambia. AIDS. 2013;27(1):93–103. 10.1097/QAD.0000000000000039 PMC4070372 24088689

[23] Blumenthal P.D, Voedisch A, Gemzell-Danielsson K. Strategies to prevent unintended pregnancy: increasing use of long-acting reversible contraception. Human Reproduction Update. 2010;17(1):121–37. doi: 10.1093/humupd/dmq026 20634208 10.1093/humupd/dmq026

